# Comparison of Efficacy and Safety Profile of Sodium-Glucose Cotransporter-2 Inhibitors as Add-On Therapy in Patients With Type 2 Diabetes

**DOI:** 10.7759/cureus.14268

**Published:** 2021-04-03

**Authors:** Mazhar Hussain, Asim Elahi, Javed Iqbal, Muhammad Bilal Ghafoor, Habib Rehman, Shoaib Akhtar

**Affiliations:** 1 Pharmacology, Sheikh Zayed Medical College and Hospital, Rahim Yar Khan, PAK; 2 Internal Medicine, Pikeville Medical Center, Pikeville, USA; 3 Medicine, Sheikh Zayed Medical College and Hospital, Rahim Yar Khan, PAK; 4 Pathology, Sheikh Zayed Medical College and Hospital, Rahim Yar Khan, PAK

**Keywords:** sodium-glucose cotransporter-2 (sglt-2) inhibitors, glycated hemoglobin (hba1c), body mass index (bmi), efficacy, safety profile, adverse effects

## Abstract

Background

Type 2 diabetes is a chronic metabolic disorder that is escalating at an alarming rate worldwide. Sodium-glucose cotransporter-2 (SGLT-2) inhibitors are recent oral antihyperglycemic drugs (OADs) with a unique mechanism of action.

Objectives

This study aimed compared the efficacy and safety profiles of two SGLT-2 inhibitors, empagliflozin and dapagliflozin, in patients with type 2 diabetes as add-on therapy to traditional first-line OADs.

Methods

We conducted a randomized controlled trial comparing empagliflozin and dapagliflozin in patients with type 2 diabetes. Patients were included in the study if they had type 2 diabetes with inadequate glycemic control, defined as glycated hemoglobin (HbA1c) of 7.5% to 11.0%, treated with conventional first-line OADs. Study participants were randomly assigned into two groups. Group A patients received oral empagliflozin, 10 to 25 mg, and Group B patients received oral dapagliflozin, 5 to 10 mg, for 12 weeks. The primary endpoint was the efficacy profile for each SGLT-2 agent in terms of body weight changes, body mass index (BMI), fasting blood glucose (FBG), and HbA1c. The secondary endpoint was to determine the safety and tolerability profiles of each SGLT-2 agent.

Results

After 12 weeks of treatment, the mean body weight was reduced significantly in both groups from baseline (empagliflozin: -3.2 kg ± 5.5 kg, p = 0.003; dapagliflozin -2.1 kg ± 4.6 kg, p = 0.008). However, the mean body weight reduction between groups was not statistically significant (p = 0.078). BMI was significantly reduced in both groups (empagliflozin from 28.5 ± 4.9 kg/m^2^ to 25.8 ± 5.2 kg/m^2^, p = 0.002; dapagliflozin from 29 ± 5.2 kg/m^2^ to 27.7 ± 4.8 kg/m^2^, p = 0.003). However, the patients who received empagliflozin experienced a significantly greater reduction in BMI than patients who received dapagliflozin (p = 0.007).

The mean FBG was also reduced in both study groups (empagliflozin: -88.5 mg/dL ± 39.7 mg/dl, p = 0.003; dapagliflozin: -59.8 mg/dL ± 48.5 mg/dL; p = 0.007). However, the patients who received empagliflozin experienced a significantly greater reduction in mean FBG than patients who received dapagliflozin (p = 0.001). HbA1c was also significantly reduced in both groups (empagliflozin: -2.1% ± 1.1%, p = 0.002; dapagliflozin: -1.4% ± 0.9%; p = 0.004). However, patients who received empagliflozin experienced a significantly greater reduction in HbA1c than patients who received dapagliflozin (p = 0.001).

The tolerability profiles of both SGLT-2 agents were quite good, and no major adverse effects were reported in the study groups. Urinary infection occurred more often in patients who received dapagliflozin (9.3%) than in patients who received empagliflozin (4.5%; p = 0.002). Patients in the dapagliflozin group also had a higher incidence of genital infections (7.3%) than those in the empagliflozin group (3.8%; p = 0.001).

Conclusion

Both empagliflozin and dapagliflozin demonstrated excellent efficacy and safety profiles in our study. These agents should be considered as add-on therapy in patients with type 2 diabetes taking conventional first-line OADs.

## Introduction

Type 2 diabetes is a chronic metabolic disorder with an escalating incidence worldwide, found in nearly one in 11 people [1]. The number of diabetes cases worldwide is expected to rise from 450 million to 642 million in 20 years. Pakistan has the fourth-most diabetes cases globally, and in 2019, 19.4 million people in Pakistan had diabetes; the number of cases is projected to reach 26.2 million in 2030 and 37.1 million in 2045 [2]. This level of prevalence of diabetes will add significant morbidity and mortality and pose an enormous economic burden on national resources [1-2].

The treatments available for patients with diabetes have seen significant advancements. Oral antihyperglycemic drugs (OADs) are usually first-line therapies and lifestyle changes for patients with type 2 diabetes. There are currently seven first-line OADs, with several more in the development and the approval stages. The current class of OADs consists of biguanides, sulphonylurea, alpha glycosidase inhibitors, thiazolidinediones, glucagon-like peptide-1 (GLP-1) receptor agonists, dipeptidyl peptidase-4 (DPP-4), and sodium-glucose cotransporter-2 (SGLT-2) inhibitors. The antihyperglycemic effects of these drugs are mediated through various mechanisms [3].

SGLT-2 inhibitors are the latest OADs with a unique mechanism of action. The insulin-independent anti-hyperglycemic effect of SGLT-2 inhibitors is mediated by suppressing the glucose reabsorption in renal tubules, facilitating its excretion in urine. SGLT-2 inhibitors are optimal in this scenario. Given that approximately 90% of the filtered load of glucose is reabsorbed in the kidney's proximal convoluted tubule, SGLT-2 inhibitors are an innovative approach to reducing glycemia. The United States (US) Food and Drug Association (FDA) has approved three drugs in this class: canagliflozin, dapagliflozin, and empagliflozin [4-5].

SGLT-2 inhibitors have excellent efficacy, safety, and tolerability profiles without significant risk of hypoglycemia [6]. Beyond improving glycemic control, SGLT-2 inhibitors offer pleiotropic effects on body weight, blood pressure, hyperuricemia, dyslipidemia, and fatty liver disease [7]. Clinical trials conducted in the US and Europe have shown favorable SGLT-2 safety in cardiovascular and kidney disease [8-9]. However, in Asia, data are limited, and in Pakistan, no study has been conducted to assess the cardiovascular safety profile of SGLT-2 inhibitors. SGLT-2 inhibitors are generally reserved as a second or third-line antihyperglycemic drug in the treatment of type 2 diabetes, but they can also be used as monotherapy when metformin is contraindicated [10].

The goal of this study was to assess the 12-week safety and tolerability profile of dapagliflozin and empagliflozin as add-on therapy in patients with type 2 diabetes currently being treated with conventional first-line OADs.

## Materials and methods

We conducted a 12-week randomized, controlled trial at five private clinics and the diabetes clinic of Sheikh Zayed Hospital. A total of 615 patients were recruited from these clinical settings, and 342 patients met the inclusion criteria and were enrolled in the study. To be included in the study, patients had to have type 2 diabetes with inadequate glycemic control defined as glycated hemoglobin (HbA1c) of 7.5% to 11% treated with different first-line OAD combinations, such as metformin, pioglitazone, glibenclamide, glimepiride, sitagliptin, and vildagliptin. The study excluded patients with a history of type 1 diabetes, ketoacidosis, significant hepatic disease, cardiovascular disease, unstable/rapidly progressing renal disease, malignancy, pancreatic disorders, genitourinary infection, and steroid use. All study participants provided informed written consent for inclusion in the study. The study protocol was approved by the Institutional Review Board of Sheikh Zayed Medical College and Hospital.

The study population was divided into two groups via random sampling. Group A patients were given once-daily oral empagliflozin, 10 mg to 25 mg, and patients in Group B were given once-daily dapagliflozin, 5 mg to 10 mg, as an add-on therapy for 12 weeks. The doses of SGLT-2 inhibitors, dipeptidyl peptidase-4 (DPP-4) inhibitors, thiazolidinediones, and metformin were maintained during the study, while sulphonylurea doses were reduced if patients developed hypoglycemic episodes.

The study's primary endpoint assessed each drug's efficacy by measuring body weight changes, body mass index (BMI), fasting blood glucose (FBG), and HbA1c from baseline. The secondary endpoint was determining each drug's safety and tolerability profiles by assessing adverse effects from baseline. All the parameters were measured before starting SGLT-2 inhibitor therapy and at the end of the study. FBG was analyzed via the glucose oxidase peroxidase method. HbA1c was measured by liquid chromatography, while fasting serum lipid profile was measured by the enzymatic endpoint method.

Data analysis

Data were analyzed using the Statistical Package for Social Sciences (SPSS) for Windows, version 16.0 (SPSS, Inc., Chicago, IL). The values of numeric data were expressed as mean ± standard deviation (SD). The frequency data were expressed as a percentage. The difference in the primary endpoint from baseline was analyzed using the paired t-test, while the difference in secondary endpoints from baseline was determined via the Chi-square test. We considered p < 0.05 as statistically significant and p < 0.01 as highly significant.

## Results

**Figure 1:**
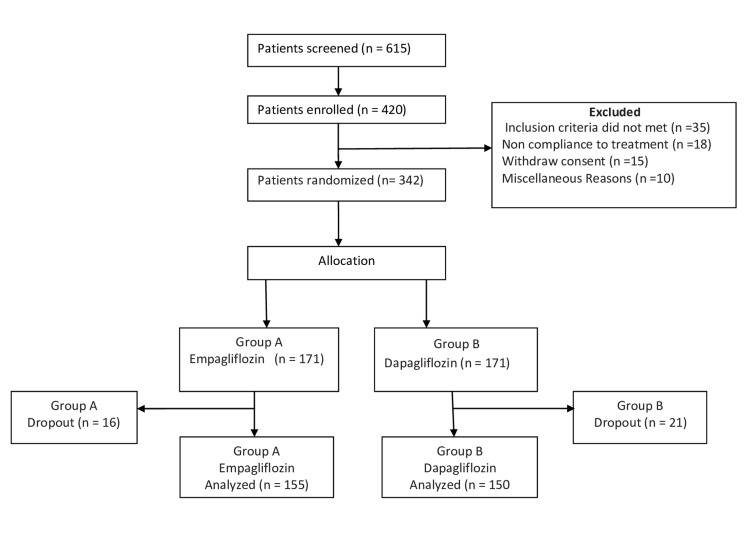
Study Flow Chart

A total of 615 patients with type 2 diabetes were recruited, of whom 420 were considered for the study. Seventy-eight patients were excluded, leaving a total of 342 patients included in the study, randomized into two groups. Sixteen patients in the empagliflozin group and 21 patients in the dapagliflozin group were dropped out. One hundred fifty-five patients received empagliflozin, and 150 patients received dapagliflozin (Figure 1).

There was no difference in the baseline demographic characteristics and clinical study parameters in both study groups at the start of the study. The number of patients taking other OADs is presented in Table 1.

**Table 1:** Baseline Characteristics, Clinical Parameters, and Concomitant Therapies BMI: body mass index; DPP-4: dipeptidyl peptidase-4; FBG: fasting blood glucose; HbA1c: glycated hemoglobin; HDL: high-density lipoprotein; LDL: low-density lipoprotein; OAD: oral antihyperglycemic drug; SD: standard deviation

Patient Characteristics	Empagliflozin (n = 155)	Dapagliflozin (n = 150)	P-value
Age (years)	52.5 ± 15.6	49.4 ± 18.2	0.63
Gender (M/F)	109 (70%)/46 (30%)	100 (67%)/50 (33%)	0.32
Body weight (kg)	93.5 ± 18.4	95.6 ± 21	0.42
BMI (kg/m^2^)	28.5 ± 4.9	29 ± 5.2	0.96
Diabetes duration (years)	9.2 ± 3.8	8.9 ± 5.6	0.72
Clinical Parameters (mean ± SD)			
FBG (mg/dL)	188 ± 36.5	195 ± 42	0.81
HbA1c (%)	10.1 ± 3.4	9.5 ± 2.8	0.36
Total Cholesterol (mg/dL)	164 ± 32.5	142 ± 26.5	0.52
Triglycerides (mg/dL)	187 ± 29.7	190 ± 35.6	0.71
LDL Cholesterol (mg/dL)	98 ± 32	108 ± 18.5	0.42
HDL Cholesterol (mg/dL)	40.5 ± 8.6	43 ± 7.8	0.64
Concomitant OAD (N (%))			
Biguanides	21 (13.5%)	22 (14.6%)	0.42
Thiazolidinediones	3 (2%)	5 (3.3%)	0.71
DPP-4 Inhibitors	10 (6.4%)	12 (8%)	0.32
Sulphonylurea	16 (10.3%)	11 (7.3%)	0.66
Sulphonylurea + Biguanides	38 (24.5%)	42 (28%)	0.28
Sulphonylurea + Thiazolidinediones	10(6.4%)	9 (6%)	0.39
DPP-4 Inhibitors + Biguanides	40 (25.8%)	35 (23.3%)	0.64
Thiazolidinediones + Biguanides	17 (11%)	14 (9.3%)	0.34

After 12 weeks of treatment, the mean body weight was reduced significantly in both groups. Patients receiving empagliflozin had a mean body weight reduction from baseline of 3.2 kg ± 5.5 kg (p = 0.003), and patients receiving dapagliflozin had a mean body weight reduction of 2.1 kg ± 4.6 kg (p = 0.008). The difference in mean body weight between patients receiving empagliflozin and patients receiving dapagliflozin at the end of the study was not statistically significant (p = 0.078). BMI was significantly reduced in both groups (empagliflozin from 28.5 ± 4.9 kg/m^2^ to 25.8 ± 5.2 kg/m^2^, p = 0.002; dapagliflozin from 29 ± 5.2 kg/m^2^ to 27.7 ± 4.8 kg/m^2^, p = 0.003). However, the patients who received empagliflozin experienced a significantly greater reduction in BMI than patients who received dapagliflozin (p = 0.007).

Similarly, the mean FBG was significantly reduced in both study groups. Patients receiving empagliflozin had a mean FBG reduction of 88.5 mg/dL ± 39.7 mg/dL (p = 0.003). Patients receiving dapagliflozin had a mean FBG reduction of 59.8 mg/dL ± 48.5 mg/dL (p = 0.007). However, patients who received empagliflozin experienced a significantly greater reduction in mean FBG than patients who received dapagliflozin (p = 0.001) after 12 weeks of treatment.

The mean HbA1c was also significantly reduced in both groups. Patients who received empagliflozin had a mean HbA1c reduction by 2.1% ± 1.1% (p = 0.002), and patients who received dapagliflozin had a mean HbA1c reduction by 1.4% ± 0.9% p = 0.004). Patients who received empagliflozin experienced a significantly greater reduction in the mean HbA1c than patients who received dapagliflozin (p = 0.001) after 12 weeks of treatment (Table 2).

**Table 2:** Changes From Baseline in Both Study Groups † Differences within groups measured at baseline and week 12 ‡ Differences within the group measured at baseline and week 12 BMI: body mass index; FBG: fasting blood glucose; HbA1c: glycated hemoglobin

Group A Empagliflozin (n = 128)				Group B Dapagliflozin (n = 127)				P-value‡
Parameters	0 weeks	12 weeks	P-value†	Parameters	0 weeks	12 weeks	P-value†	
Body weight (kg)	93.5 ± 18.4	89.5 ± 16.5	0.003	Body weight (kg)	95.6 ± 21	93.4 ± 17	0.008	0.078
BMI (kg/m^2^ )	28.5 ± 4.9	25.8 ± 5.2	0.002	BMI (kg/m^2^)	29 ± 5.2	27.7 ± 4.8	0.003	0.007
FBG (mg/dL)	188 ± 36.5	132.8 ± 48.5	0.003	FBG (mg/dL)	195 ± 42	151 ± 38.5	0.007	0.001
HbA1c (%)	10.1 ± 3.4	7.5 ± 2.2	0.002	HbA1c (%)	9.5 ± 2.8	8.2 ± 3.2	0.004	0.001

Both drugs' tolerability profiles were quite good, and no major adverse effects were reported in either study group. However, minor adverse effects were observed in both study groups (Table 4).

**Table 3:** Adverse Effects

Adverse Effects	Group A Empagliflozin (n = 155)	Group B Dapagliflozin (n = 150)	P-value*
Deaths	0	0	
Adverse effect leading to discontinuation	5 (3.2%)	8 (5.3%)	0.66
Documented hypoglycemia (glucose < 70 mg/dL)	4 (2.6%)	6 (4%)	0.48
Hypersensitivity	2 (1.3%)	3 (2%)	0.36
Hypotension/dehydration/hypovolemia	3 (2.0%)	4 (2.6%)	0.25
Frequency/nocturia (three times/night)	10 (6.4%)	13 (8.6%)	0.32
Urinary tract infection	7 (4.5%)	14 (9.3%)	0.002
Genital infection	6 (3.8%)	11 (7.3%)	0.001
Total adverse effects	37 (23.8)	59 (39.3)	0.001

Urinary infections occurred more often in patients who received dapagliflozin (9.3%) than in patients who received empagliflozin (4.5%; p = 0.002). Patients in the dapagliflozin group also had a higher incidence of genital infections (7.3%) than those in the empagliflozin group (3.8%; p = 0.001).

## Discussion

The present study was conducted to determine the efficacy and safety profiles of empagliflozin compared with dapagliflozin as an add-on therapy in patients with type 2 diabetes experiencing inadequate glycemic control with conventional first-line OADs. Both empagliflozin and dapagliflozin have excellent efficacy and safety profiles. However, empagliflozin caused a more significant improvement in mean body weight, FBG, and HbA1c with fewer adverse effects than dapagliflozin.

The pharmacologic management of diabetes changes over time due to its progressive nature. This makes achieving optimal glycemic control with monotherapy a challenge and eventually causes patients to need two to four OADs as combination therapy, as recommended by both the American Diabetes Association (ADA) and the European Association for the Study of Diabetes (EASD) [11].

In our study, both empagliflozin and dapagliflozin significantly reduced body weight and HbA1c. These results support the findings reported in another study conducted in two different cohorts at 12 and 24 weeks where empagliflozin produced similar reductions in body weight and HbA1c [12]. Similarly, another clinical study reported that dapagliflozin reduced HbA1c and body weight over 152 weeks in patients with inadequate glycemic control via metformin [13]. Both studies compared the SGLT-2 inhibitor against a placebo control group, while our study compared the outcomes of two SGLT-inhibitors against each other.

Our results were similar to those of a 52-week study by Ku et al., who reported that empagliflozin reduced body weight, blood glucose levels, and HbA1c to a greater degree than dapagliflozin [14]. Ku et al. also reported that empagliflozin improved cardiometabolic risk factors more significantly than dapagliflozin and had a low incidence of genitourinary infection.

A review regarding the efficacy, safety, and tolerability of different SGLT-2 inhibitors demonstrated that empagliflozin is one of the safer choices and can be prescribed in patients with type 2 diabetes with renal impairment (a parameter that our study did not explore) [15].

A study conducted in the United Arab Emirates reported that canagliflozin (300 mg), in combination with metformin for 26 weeks, provided a greater reduction in HbA1c (-0.79%) than empagliflozin 25 mg (-0.64%) and dapagliflozin 10 mg (-0.41%) [16]. A study conducted in China reported that empagliflozin and dapagliflozin reduced body weight, FBG, and HbA1c for patients with type 2 diabetes, similar to our results [17]. Also, both drugs ameliorated hepatic dysfunction and improved insulin resistance over six months [17].

The efficacy, safety, and tolerability profile of empagliflozin and dapagliflozin as an add-on therapy were investigated in various clinical studies that yielded similar results to our study [18-26]. Both drugs reduced body weight and provided excellent glycemic control with no risk of severe hypoglycemia. Moreover, the risk of urogenital infection varied from 1% to 9%, which is similar to the risk we found.

The risk of atherosclerotic disease is very high in patients with type 2 diabetes. Treatment with dapagliflozin provides cardiovascular safety with a low rate of cardiovascular death and hospitalization due to heart failure [27]. Empagliflozin also reduces cardiovascular events and delays kidney disease progression in patients with type 2 diabetes with cardiovascular comorbidities. A systematic review and meta-analysis of 27 studies also showed that SGLT-2 inhibitors reduced the risk of renal and cardiovascular disease impairment in patients with chronic kidney disease and patients with diabetes [28].

## Conclusions

Both empagliflozin and dapagliflozin have excellent efficacy, safety, and tolerability profiles. They can be safely used as an add-on therapy to conventional OADs in patients with type 2 diabetes. There is a further need to explore the efficacy and safety of SGLT-2 inhibitors in diabetic patients with cardiovascular disease and renal impairment. Moreover, a study with a larger sample size and longer duration is warranted to confirm the safety and tolerability profile.
